# Down-regulation of GRP78 is associated with the sensitivity of chemotherapy to VP-16 in small cell lung cancer NCI-H446 cells

**DOI:** 10.1186/1471-2407-8-372

**Published:** 2008-12-17

**Authors:** Yingyan Wang, Wei Wang, Siyan Wang, Jiarui Wang, Shujuan Shao, Qi Wang

**Affiliations:** 1 Diagnostics Laboratory Center, Dalian Medical University, Dalian, Liaoning, PR China; 2 Department of Respiratory Medicine, The Third People's Hospital of Dalian, Dalian, Liaoning, PR China; 3 Department of Emergency Medicine, The Second Hospital Affiliated to Dalian Medical University, Dalian, Liaoning, PR China; 4 Postgraduate School, Dalian Medical University, Dalian, Liaoning, 116044, PR China; 5 Department of Histology and Embryology, Dalian Medical University, Dalian, Liaoning, PR China; 6 Department of Respiratory Medicine, The Second Hospital Affiliated to Dalian Medical University, Dalian, Liaoning, PR China

## Abstract

**Background:**

Chemotherapy resistance remains a major obstacle for the treatment of small cell lung cancer (SCLC). Glucose-regulated protein 78 (GRP78), an endoplasmic reticulum chaperone, plays a critical role in chemotherapy resistance in some cancers. However, whether the suppression of the chaperone can enhance the sensitivity of chemotherapy in SCLC is still unclear.

**Methods:**

The SCLC NCI-H446 cells were divided into three groups: BAPTA-AM→A23187-treated group, A23187-treated group and control-group. Immunofluorescence, western blot and RT-PCR were used to assess the expression of GRP78 at both protein and mRNA levels. Cell apoptosis and the cell cycle distributions of the cells were analyzed by flow cytometry in order to evaluate the therapeutic sensitivity to VP-16.

**Results:**

The expression of GRP78 at both protein and mRNA levels in the BAPTA-AM→A23187-treated cells dramatically decreased as compared to that in both A23187-treated and control groups. After treatment by VP-16, the percentage of apoptotic cells in BAPTA-AM→A23187-treated cells were: 33.4 ± 1.01%, 48.2 ± 1.77%, 53.0 ± 1.43%, 56.5 ± 2.13%, respectively, corresponding to the concentrations of BAPTA-AM 10, 15, 25, 40 μM, which was statistically significant high in comparison with the A23187-treated group and untreated-group (7.18 ± 1.03% and 27.8 ± 1.45%, respectively, p < 0.05). The results from analysis of cell cycle distribution showed that there was a significantly decreased in G_1 _phase and a dramatically increased in S phase for the BAPTA-AM→A23187-treated cells as compared with the untreated cells.

**Conclusion:**

BAPTA-AM is a strong inhibitor of GRP78 in the NCI-H446 cell line, the down-regulation of GRP78 can significantly increase the sensitivity to VP-16. The suppression of GRP78 may offer a new surrogated therapeutic approach to the clinical management of lung cancer.

## Background

Lung cancer is currently the leading cause of cancer deaths worldwide no matter in men or women [1]. Small cell lung cancer (SCLC) accounts for 13%–15% of all lung cancer worldwide [2]. Chemotherapy is an important means of the treatment for patients with SCLC. However, the drug resistance as developed during the treatment really limits the efficacy of chemotheraspy. Multiple pathways are suggested to be involved in the complexity of chemotherapy resistance in SCLC. A favorable mechanism for explaining the chemotherapy resistance is speculated as the presence of microenvironment conditions, glucose starvation and hypoxia that occur naturally in solid tumors [3]. Cells respond to these stressful conditions through the synthesis of a kind of evolutionarily conserved protein, named as glucose-regulated proteins (GRPs) [4], which are known to show the protective role as a molecular chaperone against endoplasmic reticulum (ER) stress-induced cell death in mammalian cells [5-7]. GRP78/BiP, a well-characterized GRP member with molecular weight of 78 kda, belongs to the highly conserved heat shock protein 70 (HSP70) family, resides primarily in ER of mammalian cells [8,9]. It can be regulated by several cellular stresses which perturb ER function and homeostasis including some inhibitors and inducers [10]. Generally, the commonly used inducers are 2-deoxyglucose, tunicamycin and calcium ionophore A23187; the commonly used inhibitors are thapsigargin and membrane-permeant Ca^2+ ^chelator BAPTA-AM [11,12]. A line of studies have shown that GRP78 plays a protective role in maintaining cell viability against several kinds of stress in a variety of cancers [13-15]. In our recent study, we demonstrated that the overexpression of GRP78 under the induction of A23187 is associated with chemotherapy resistance to VP-16 in human lung cancer [16,17]. Thus, increasing attention on the role of GRP78 plays in chemotherapy resistance during therapy has been brought. However, most of the reports focus on the up-regulation of GRP78, while whether the suppression of GRP78 could enhance the sensitivity of chemotherapy in cancer still remains unclear. Herein, we intended to investigate the down-regulation of GRP78 by BAPTA-AM, and the function of the suppression in the resistance to VP-16 in SCLC NCI-H446 cells.

## Methods

### Cell culture and treatment

The NCI-H446 cell line was obtained from the American Type Culture Collection (Manassas, VA, USA) and cultured in RPMI-1640 medium (Sigma-Aldrich Co, St. Louis, MO, USA) supplemented with 5% fetal bovine serum (FBS) and 100 μg/ml kanamycin at 37°C in a humidified atmosphere containing 5% CO_2 _and 95% air. The medium was routinely changed 3 days after seeding. All experiments were performed using exponentially growing cells and repeated at least 3 times. The cells were divided into BAPTA-AM→A23187-treated group, A23187-treated group and control-group. For BAPTA-AM→A23187-treated group, the cells were exposed to BAPTA-AM (sigma, St. Louis, MO) at different concentrations of 10,15, 25, and 40 μM, respectively for 2 h before the addition of A23187 (Sigma Chemical Co, Taufkirchen, Germany) at the concentration of 2 μM for 24 h; For A23187-treated group, the cells were added A23187 alone at 2 μM for 24 h; For control-group, the cells were cultured in medium for 24 h. Cell survival to VP-16 (Sigma, St. Louis, MO, USA) was determined by flow cytometry. Briefly, following exposure to BAPTA-AM or A23187, the cells of the three groups were incubated with VP-16 at concentration of 30 μM for 6 h, respectively, then, the cells were cultured in new media for another 48 h further before the harvest for the analysis of apoptosis and cell cycle using flow cytometry (FAC star; BD Biosciences).

### RNA isolation and conventional RT-PCR

Total RNA was extracted from the cells with Trizol (GIBCO BRL, Gaithersburg, MD, USA) according to the manufacturer's instructions. In brief, the lysis of the cells in Trizol was centrifuged at 10000 g at 4°C for 15 min in the presence of chloroform. The upper aqueous phase was collected and the RNA was precipitated by addition of 100% isopropanol and a high salt precipitation solution (0.8 M sodium citrate and 1.2 M NaCl) and centrifuged at 7500 g at 4°C for 5 min. RNA pellets were washed with ice-cold 75% ethanol, dried, resuspended in sterile water and quantified by spectrometry. RT-PCR was performed with an RNA PCR Kit (AMV, Ver.3.0, TaKaRa, Japan) according to the manufacturer's instructions. The housekeeping gene, β-actin was used as an internal control to confirm equal loading in each experiment and was amplified from the same cDNAs. The primers specific for GRP78 and β-actin are shown in Table 1. Reverse transcription was performed in a 10 μl volume mixture made up of 1 μl (≤1 μg) of total RNA, 0.5 μl Oligo dT-Adaptor primer (2.5 pmol/μl), 1 μl of 10 – Reverse-transcription buffer, 1 μl dNTP mixture (10 mM each), 0.5 μl AMV Reverse transcriptase XL (5 ^U^/μl) and 0.25 μl RNA ribonuclease inhibitor (40 ^U^/μl). Reverse transcription was performed at 42°C for 30 min and terminated at 95°C for 5 min. The PCR reaction was performed in 10 μl of 5 – PCR buffer, 0.25 μl Taq DNA polymerase (5 ^U^/μl), 10 μl RT product and 0.5 μl of each primer (20 pmol/μl). The PCR conditions were as follows: 1 cycle of 94°C for 4 min, 30 cycles of 94°C for 1 min, 58°C for 30 sec, 72°C for 30 sec and 72°C for 10 min. A total of 8 μl PCR products was separated on a 1.5% agarose gel and stained with ethidium bromide for visualization. The relative abundance of each PCR product was determined by quantitative analysis of digital photographs of gels using Labworks 4.6 software (UVP Products, Upland, CA, USA). The integral optical density (IOD) values of GRP78 and the β-actin were measured. The ratio of IOD_GRP78_/IOD_β-actin _was used to express the relative level of GRP78 mRNA.

**Table 1:** Characteristics of primers used for conventional RT-PCR

Genes	Sequence of primers	Products(bp)
GRP78	5' GATAATCAACCAACTGTTAC 3' 5' GTATCCTCTTCACCAGTTGG 3'	577
β – actin for GRP78	5' TCGTCACCAACTGGGACGACATGG 3' 5' GATCTTGATCTTCATTGTGCTGGG 3'	750

### Immunofluorescence

After treatment, the cells were washed twice with PBS, fixed with 4% paraformaldehyde in PBS for 10 min, permeablized in PBS containing 0.1% Triton X-100 and 5% bovine serum albumin for 30 min before the detection of GRP78 with immunofluorescence. The media with anti-GRP78 (C-20) goat polyclonal antibody (Santa Cruz Biotechnology, Inc., Santa Cruz, CA) at a dilution of 1:1000 were introduced into the culture chamber and the cells were incubated at 4°C over night. After being washed with PBS for three times, the cells were stained with anti-goat FITC green-conjugated secondary antibody (Santa Cruz Biotechnology, Inc) at a dilution of 1:100 at 37°C for 30 min. Cell images were subsequently captured with fluorescence microscope and analyzed using Northern exposure image analysis/archival software (Mississauga, Ontario, Canada).

### Protein isolation and Western blot

Cells were collected and centrifuged at 4000 rpm for 5 min, the supernatants were discarded before ice-cold RIPA buffer (PBS, pH 7.4 containing 1% NP_40_, 0.5% sodium deoxycholate, 0.1% SDS with freshly added 10 mg/ml Phenylmethanesulphonyl fluoride) was added to the cell pellets. After a 30 min incubation on ice, samples were spun at 10444 rpm for 10 min at 4°C and the supernatants were collected. Two additional centrifugations were performed to produce clarified lysates. Protein concentrations of the resulting lysates were determined by Coomassie Brilliant Blue staining. An equivalent of 50 μg protein in each sample was denatured at 90°C for 5 min, loaded on a 10% SDS-polyacrylamide gel containing a 4% stacking gel, together with a molecular weight standard (Gibco BRL, Gaithersburg, MD, USA), and then electrophoresed at 180 V for 1.5 h in Tris-glycine running buffer (25 mml/L Tris-base, 250 mmol/L glycine, 0.1% SDS). Proteins were electroblotted onto a nitrocellulose membrane. The blotted membrane was blocked with 5% nonfat dry milk in PBST (PBS, pH 7.4, Tween 20) with gentle shaking at room temperature overnight and then incubated for 2 h at room temperature in 1% bovine serum albumin (BAS)/PBST containing goat-anti-GRP78 polyclonal antibodies (Santa Cruz Biotechnology, Santa Cruz, CA, USA, 1:400) with gentle shaking. Following being washed in PBST for three times, the membrane was then incubated for 1 h at room temperature in 1% BSA/PBST containing the second antibody (rabbit antigoat IgG, Santa Cruz Biotechnology, 1:1000) with gentle shaking. The membrane was washed again for three times in PBST. Goat-anti-β-actin polyclonal antibody (43 kDa, Santa Cruz Biotechnology, 1:500) was used as an internal control. Rabbit anti-goat secondary antibody (1:1000) was used subsequently. GRP78 protein expression was detected using an ECL (enhanced chemiluminescence) Plus kit (Amersham Biosciences, Piscataway, NJ, USA). Signals were visualized by autoradiography and determined by quantitative analysis of digital photographs of gels using Labworks 4.6 software (UVP Products, Upland, CA, USA). The integral optical density (IOD) values of GRP78 and β-actin were measured. The ratio of IODGRP78/IOD β-actin was used to express the relative level of GRP78 protein.

### Analysis of apoptosis

After the treatment with VP-16, the cells were trypsinized, washed with ice-cold PBS (pH 7.4) for two times and re-suspended in 1 × binding buffer (10 mM HEPES, pH 7.4, 140 mM NaCl, 2.5 mM CaCl_2_) at a concentration of 1 × 106 cells/ml. 100 μl of cell suspension was transferred to 5 ml plastic tubes, 5 μl of annexin V-fluorescein isothiocyanate (PharMingen) and 5 μl propidium iodide (PI) were added. The cells were gently vortexed and incubated in the dark at room temperature for 25 min. 400 μl of binding buffer was added to each tube, and annexin V staining was analyzed within 1 h by flow cytometry (FAC star; BD Biosciences). Cells negatively stained by both PI and annexin V are considered as live cells, annexin V positive stained cells are considered as the early apoptotic cells, and both PI and annexin V positive stained cells are primarily cells in late stages of apoptosis. The experiments were repeated two to three times.

### Cell cycle analysis

Following seeding, the VP-16-treated cells were trypsinized and fixed in 70% ethanol. The fixed cells were treated with PBS containing 0.1% (v/v) Triton X-100, 0.2 mg/ml DNase-free RNase, and 20 g/ml PI for 30 min at room temperature. The cell cycle distributions were analyzed by a fluorescence-activated cell sorting (FACS) scan flow cytometry analysis (FAC star; BD Biosciences) and data were analyzed with ModFit software. The cell cycle distribution measurements were repeated three to five times.

### Statistical analysis

The statistical analyses were carried out with SPSS 12.0 for Windows software. Student's t-test and χ^2 ^test (chi-square test) were used to compare the results of flow cytometry, expression of GRP78 mRNA and GRP78 protein respectively. A value of p < 0.05 was regarded as statistically significant.

## Results

### Down-regulation of GRP78 mRNA by BAPTA-AM

**Figure 1:**
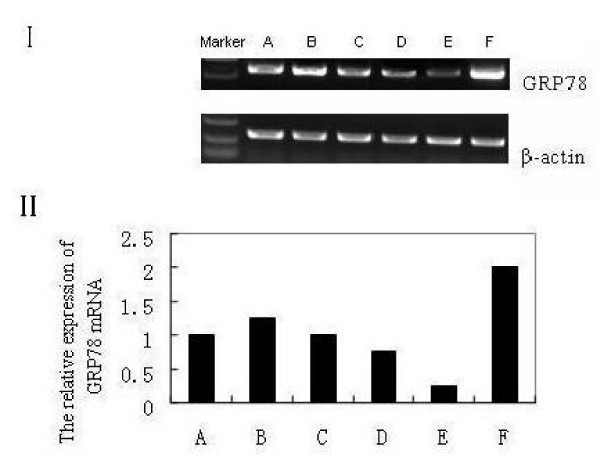
**The results of GRP78 RT-PCR products for the NCI-H446 cells**. (A): untreated (control) cells; (B-E): cells treated with BAPTA-AM at 10 μM (B), 15 μM (C), 25 μM (D), 40 μM (E) for 2 h respectively, prior to the addition of A23187 at 2 μM for 24 h; (F): cells treated with 2 μM A23187 alone for 24 h. I: Electrophoregram II: Bar graph.

To examine the effect of BAPTA-AM on the expression of GRP78 mRNA in NCI-H446 cell line, we carried out a conventional RT-PCR analysis on the cells in BAPTA-AM→A23187-treated group, A23187-treated group and control group. When the cells were exposed to BAPTA-AM alone at different concentrations (10, 15, 25, and 40 μM) for 2 h, the signals of GRP78 mRNA were too weak to be assayed easily with an approximately concentration-depended manner (date not shown), whereas after the addition of A23187 at the concentration of 2 μM for 2 h, the GRP78 mRNA signals of the BAPTA-AM pre-treated cells could be assayed obviously. Therefore, the cells were grouped three as above. As shown in Fig. 1, the expression of GRP78 mRNA in BAPTA-AM→A23187-treated group was decreased greatly (around 2–4-folds) compared with that of A23187-treated and control groups, also shown a nearly BAPTA-AM concentration-dependent manner.

### Down-regulation of GRP78 protein by BAPTA-AM

**Figure 2:**
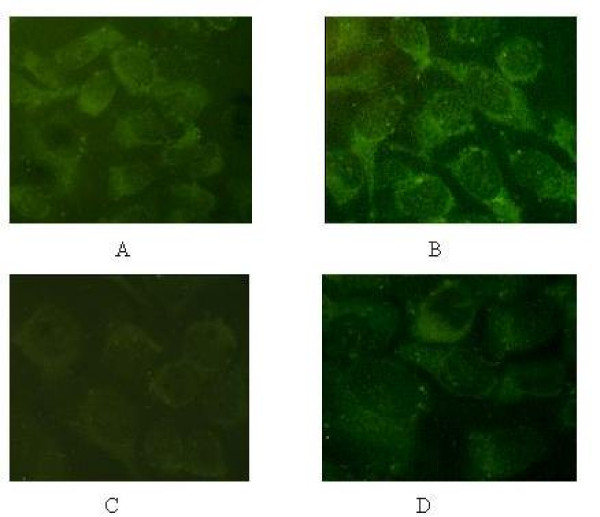
**The expression of GRP78 at the protein level detected by Immunofluorescence in NCI-H446**. (A): untreated (control) cells; (B): cells treated with 2 μM A23187 alone for 24 h; (C): cells pretreated with BAPTA-AM at 40 μM for 2 h prior to the addition of A23187 at 2 μM for 24 h. (D): cells treated with BAPTA-AM at 10 μM alone for 2 h.

**Figure 3:**
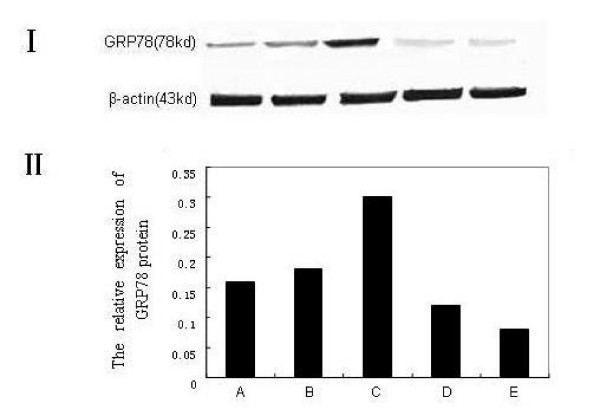
**The expression of GRP78 at the protein level by western blots in NCI-H446**. (A): untreated (control) cells; (B): cells treated with BAPTA-AM at 10 μM alone for 2 h; (C): cells treated with A23187 at 2 μM alone for 24 h; (D-E): cells treated with BAPTA-AM at 25 μM (D), 40 μM (E) for 2 h respectively, prior to the addition of A23187 at 2 μM for 24 h.I: Electrophoregram II: Bar graph.

To analyze whether the expression of GRP78 protein was related to the GRP78 mRNA for the cells pretreated with BAPTA-AM, we carried out an immunofluorescence and western blots assay on the NCI-H446 cells. The result of immunofluorescence showed that GRP78 displayed a perinuclear, reticular pattern of distribution in the cells (Fig. 2). The expression of GRP78 in BAPTA-AM→A23187-treated group was obviously weak as compared to the A23187-treated and control groups. The results from the Western blot analysis agreed well with that from immunofluorescence and the RT-PCR (Fig. 3). As shown in Fig. 3, the expression of GRP78 protein in BAPTA-AM→A23187-treated group was decreased greatly (around 3-folds) compared with that of A23187-treated group and control group, with a character of BAPTA-AM concentration-dependent.

### Effect of BAPTA-AM pretreatment on cell resistance to VP-16

**Figure 4:**
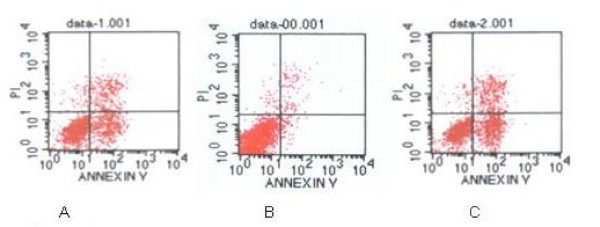
**Measurement of apoptotic cells under VP-16 (30 μM) treatment for 6 h determined by FACS analysis**. (A): untreated (control) cells; (B): cells treated with A23187 at 2 μM for 24 h; (C): cells pretreated with BAPTA-AM at 40 μM for 2 h prior to the addition of A23187 at 2 μM for 24 h. The cells were labeled with annexin V and PI. Viable cells were those with low annexin or no annexin and PI staining (lower left panel). Early stage apoptotic cells were represented by high annexin and low PI staining (lower right panel), later stage apoptotic cells were represented by high annexin and high PI staining (upper right panel), and necrosis cells were represented by high PI and low annexin staining (upper left panel).

To determine the relationship between the down-regulation of GRP78 and the sensitivity to anti-cancer drug VP-16 in NCI-H446 cell line, the cells were exposed to VP-16 at 30 μM for 6 h after the pretreatment with BAPTA-AM or A23187. Trypan blue exclusion assay showed less than 1% of the cells pretreated with BAPTA-AM at concentrations from 10 μM to 40 μM died (date not shown), indicating that the pretreatment with BAPTA-AM alone did not affect the cell viability. The percentage of apoptotic cells measured by flow cytometry showed that the sensitivity to VP-16 for the cells in BAPTA-AM→A23187-treated group was significantly higher as compared to both group of A23187-treated and control. As shown in Fig. 4, the percentage of apoptotic cells in BAPTA-AM→A23187-treated group were: 33.4 ± 1.01%, 48.2 ± 1.77%, 53.0 ± 1.43%, 56.5 ± 2.13% respectively, corresponding to the concentrations of BAPTA-AM at 10, 15, 25, 40 μM, respectively, which was statistically significant high in comparison with the A23187-treated group and control (7.18 ± 1.03% and 27.8 ± 1.45%, respectively, p < 0.05). Furthermore, the percentage of apoptotic cells in BAPTA-AM→A23187-treated group increased as the increment of a BAPTA-AM concentration. Together with the results of GRP78 above, it is suggested that down-regulation of GRP78 could enhance VP-16 induced apoptosis in NCI-H446 cell line.

### Cell cycle distribution

The analysis of cell cycle distributions showed that there was a significantly decrease in G_1 _phase and a dramatically increase in S phase in the BAPTA-AM→A23187-treated cells as compared with the control cells (p < 0.05, Table 2). On the contrary, there was an increased in G_1 _phase and decreased in S phase in the A23187-treated cells. Combined with the expression of GRP78, it indicated that the cell cycle distribution can be altered significantly by up- or down-regulation of GRP78 (p < 0.05), suggesting that the GRP78 mediated alteration of the sensitivity to VP-16 may be occurred through the G_1_/S arrest for the lung cancer cells.

**Table 2:** Cell cycle distribution and percentage of apoptosis for the BAPTA-AM→A23187-treated, A23187-treated, and untreated cells (x ± s) %

Groups	Cell cycle distribution			Percentage of apoptosis
		
	G_0_/G_1_	S	G_2_/M	
BAPTA-AM (40 μM) → A23187 – treated	42.00 ± 1.33	43.50 ± 2.17	14.50 ± 1.56	56.5 ± 2.13
A23187-treated (2 μM)	76.00 ± 1.24	21.40 ± 3.03	2.60 ± 2.14	7.18 ± 1.03
untreated	58.90 ± 1.48	39.00 ± 1.27	2.10 ± 1.15	27.8 ± 1.45

Compared with control (untreated) group, P < 0.05.

## Discussion

Our investigation brought up several interesting issues. First, the expression of GRP78 at both levels of the mRNA and protein was decreased significantly under the inhibition of BAPTA-AM in the NCI-H460 cell line, and the reduction of GRP78 showed a BAPTA-AM-dependent manner (Fig. 1). Secondly, the percentage of VP-16 induced cell apoptosis in the cells pretreated with BAPTA-AM was significantly higher than that in the cells pretreated with A23187 or control cells (Fig. 4). To our best knowledge, this is the first report on the down-regulation of GRP78 by BAPTA-AM, including the function of the suppression in the resistance to VP-16 in SCLC NCI-H446 cells.

GRP78 are Ca^2+^-binding proteins in the ER, the intracellular Ca^2+ ^levels have been implicated as potent regulators in the expression of the GRP78. Cytoplasmic free Ca^2+ ^is maintained at about 10-7 M by concerting actions of Ca^2+ ^pumps on ER, mitochondria and plasma membranes [18,19]. Thus, it is a common practice to employ specific calcium ionophore or Ca^2+ ^pump inhibitor to evaluate the expression of GRP78 [20]. We previously reported that a calcium ionophore, A23187, a highly potent ER stress inducer, could induce the expression of GRP78 genes and proteins in human lung cancer cell line [16]. The possible mechanism for GRP78 induction under A23187 is that it lowers the ER calcium level by releasing calcium stores, which affects protein secretion and causes accumulation of protein in the ER [21,22]. Unlike A23187, BAPTA-AM, the membrane-permeant Ca^2+ ^chelator, is a derivative of EGTA that specifically binds Ca^2+^; when modified by an acetoxymethylester group (AM) it is rendered lipophilic and thus able to cross the plasma membrane as well as intracellular membranes, including that of the ER. Once inside the cell, cellular esterases cleave the AM group, whereby the molecule is able to bind Ca^2+ ^and is no longer membrane permeant [23]. In this work, we found that BAPTA-AM attenuated the expression of GRP78 significantly. In line with our results, Juliann and Whei-meih [24,20] also respectively reported that BAPTA-AM caused a down-regulation of GRP78 in human breast cancer cell line and 9L rat brain tumor cells. However, the mechanism of BAPTA-AM caused GRP78 reduction is still not clear. Reduction of the intracellular calcium level maybe one of the possible mechanisms [23].

The apoptotic results from the cells pretreated with BAPTA-AM or A23187, prior to the addition of VP-16, showed that, the apoptotic rate of cells with lower level of GRP78 in the BAPTA-AM→A23187-treated group increase significantly as compared to A23187-treated group and control with high or normal level of GRP78, indicating that the down-regulation of GRP78 by BAPTA-AM may increase the sensitivity to VP-16 in NCI-H446 cell. Analysis of the cell cycle distributions showed that there was a great decrease in G_1 _phase and a dramatic increase in S phase for BAPTA-AM→A23187-treated group cells, suggesting that BAPTA-AM may render more sensitivity to VP-16 through the change of distribution of cell cycle in NCI-H446 cell. Nowadays, only few reports are involved on BAPTA-AM associated chemotherapy resistance. The mechanism for the chemotherapy resistance to VP-16 is complex, multiple pathways are involved. Topoisomerase IIα is an ATP-dependent nuclear enzyme that plays important roles in DNA replication and chromosome segregation by its ability to change the topological structure of DNA. VP-16 is the topoisomerase inhibitors, it can interact with the enzyme to stabilize topoisomerase-DNA complex, blocking strand-passing activity, thereby resulting in DNA breakage [25]. Furthermore, it had been proposed that the chaperone function of GRP78 could affect growth factor processing, creating a cell proliferation block to escape drug killing that only occurs in cycling cells [26]. Since VP-16 targets S phase cells [27] and here we confirmed that the inhibition of BAPTA-AM dramatically increased the percentage of S phase cells, we proposed that GRP78 might render the cells sensitive to VP-16-induced apoptosis through altering the cell cycle distribution in NCI-H446 cell line. Therefore, GRP78 suppression may become a new focal point for the cancer therapy.

## Conclusion

BAPTA-AM is a strong inhibitor of GRP78 in the NCI-H446 cell line, the down-regulation of GRP78 can increase the sensitivity to VP-16 significantly. The suppression GRP78 may offer a new therapeutic approach to the clinical management of lung cancer.

## Abbreviations

ER: endoplasmic reticulum; GRP: glucose-regulated protein; FACS: fluorescence-activated cell sorting; PI: propidium iodide; PBS: phosphate-buffered saline

## Competing interests

The authors declare that they have no competing interests.

## Authors' contributions

QW, WY and WW were responsible for the experimental design and completion of all laboratory work represented in this manuscript. SW, JW and SS participated in the design and coordination of the work involved. The manuscript was drafted by QW and YW. All authors have read and approved the final manuscript

## Pre-publication history

The pre-publication history for this paper can be accessed here:



## References

[B1] Parkin DM, Bray FI, Devesa SS (2001). Cancer burden in the year 2000. The global picture. Eur J Cancer.

[B2] Rosti G, Carminati O, Monti M, Tamberi S, Marangolo M (2006). Chemotherapy advances in small cell lung cancer. Ann Oncol.

[B3] Reddy RK, Mao C, Baumeister P, Austin RC, Kaufman RJ, Lee AS (2003). Endoplasmic reticulum chaperone protein GRP78 protects cells from apoptosis induced by topoisomerase inhibitors: role of ATP binding site in suppression of caspase-7 activation. J Biol Chem.

[B4] Lee AS (1992). Mammalian stress response: induction of the glucose-regulated protein family. Curr Opin Cell Biol.

[B5] Gething MJ (1999). Role and regulation of the ER chaperone BiP. Semin Cell Dev Biol.

[B6] Morris JA, Dorner AJ, Edwards CA, Hendershot LM, Kaufman RJ (1997). Immunoglobulin binding protein (BiP) function is required to protect cells from endoplasmic reticulum stress but is not required for the secretion of selective proteins. J Biol Chem.

[B7] Liu H, Bowes RC, Water B van de, Sillence C, Nagelkerke JF, Stevens JL (1997). Endoplasmic reticulum chaperones GRP78 and calreticulin prevent oxidative stress, Ca^2+ ^disturbances, and cell death in renal epithelial cells. J Biol Chem.

[B8] Munro S, Pelham HR (1986). An Hsp70-like protein in the ER: identity with the 78 kd glucose-regulated protein and immunoglobulin heavy chain binding protein. Cell.

[B9] Lee AS (1992). Mammalian stress response: induction of the glucose-regulated protein family. Curr Opin Cell Biol.

[B10] Li ww, Alexandre S, Cao X, Lee AS (1993). Transactivation of the grp78 promoter by Ca2+ depletion. A comparative analysis with A23187 and the endoplasmic reticulum Ca (2+)-ATPase inhibitor thapsigargin. J Biol Chem.

[B11] Chen YY, Chen G, Fan Z, Luo J, Ke ZJ (2008). GSK3beta and endoplasmic reticulum stress mediate rotenone-induced death of SK-N-MC neuroblastoma cells. Biochem Pharmacol.

[B12] Sasaya H, Utsumi T, Shimoke K, Nakayama H, Matsumura Y, Fukunaga K, Ikeuchi T (2008). Nicotine Suppresses Tunicamycin-Induced, But Not Thapsigargin-Induced, Expression of GRP78 during ER Stress-Mediated Apoptosis in PC12 Cells. J Biochem.

[B13] Gazit G, Lu J, Lee AS (1999). De-regulation of GRP stress protein expression in human breast cancer cell lines. Breast Cancer Res Treat.

[B14] Li J, Lee AS (2006). Stress induction of GRP78/BiP and its role in cancer. Curr Mol Med.

[B15] Fu Y, Lee AS (2006). Glucose regulated proteins in cancer progression, drug resistance and immunotherapy. Cancer Biol Ther.

[B16] Wang Q, Wang T, Wang Y, Wang W, Wang Y, Hu X, Shao S, Zhang J, Suo Z (2007). VP-16 Resistance in the NCI-H460 Human Lung Cancer Cell Line is Significantly Associated with Glucose-Regulated Protein78 (GRP78) Induction. Anticancer Research.

[B17] Wang YY, Wang T, Liu X, Gai H, Lin B, Wang Q (2008). The analysis of chemotherapy resistance in human lung cancer cell line with microchip-based system. Biomed Microdevices.

[B18] Rizzuto R, Brini M, Murgia M, Pozzan T (1993). Microdomains with high Ca^2+ ^close to IP3-sensitive channels that are sensed by neighboring mitochondria. Science.

[B19] Clapham DE (1995). Calcium signaling. Cell.

[B20] Chang WM, Chen KD, Chen LY, Lai MT, Lai YK (2002). Mitochondrial calcium-mediated reactive oxygen species are essential for the rapid induction of the grp78 gene in 9L rat brain tumour cells. Cellular Signal.

[B21] Gosky D, Chatterjee S (2003). Down-regulation of topoisomerase II·is caused by up-regulation of GRP78. Biochem Biophys Res Commun.

[B22] Rao RV, Peel A, Logvinova A, del Rio G, Hermel E, Yokota T, Goldsmith PC, Ellerby LM, Ellerby HM, Bredesen DE (2002). Coupling endoplasmic reticulum stress to the cell death program: role of the ER chaperone GRP78. FEBS Lett.

[B23] Grynkiewicz G, Poenie M, Tsien RY (1985). A new generation ofCa2+ indicators with greatly improved fluorescence properties. J Biol Chem.

[B24] Kiang JG, Gist ID, Tsokos GC (1998). Cytoprotection and regulation of heat shock proteins induced by heat shock in human breast cancer T47-D cells: role of [Ca^2+^]i and protein kinases. FASEB J.

[B25] Froelich-Ammon SJ, Osheroff N (1995). Topoisomerase poisons: harnessing the dark side of enzyme mechanism. J Biol Chem.

[B26] Tomida A, Tsuruo T (1999). Drug resistance mediated by cellular stress response to the microenvironment of solid tumors. Anticancer Drug Des.

[B27] Brewer JW, Hendershot LM, Sherr CJ, Diehl JA (1999). Mammalian unfolded protein response inhibits cyclin D1 translation and cell-cycle progression. Proc Natl Acad Sci USA.

